# Participatory methods in a digital setting: experiences from the co-creation of an eHealth tool for people with chronic obstructive pulmonary disease

**DOI:** 10.1186/s12911-022-01806-9

**Published:** 2022-03-18

**Authors:** Sara Lundell, Annika Toots, Pernilla Sönnerfors, Alexandra Halvarsson, Karin Wadell

**Affiliations:** 1 Department of Community Medicine and Rehabilitation, Umeå University, 901 87 Umeå, Sweden; 2 Division of Physiotherapy, Department of Neurobiology, Caring Sciences and Society, Karolinska Institutet, Huddinge, Sweden; 3 Women’s Health and Allied Health Professionals Theme, Medical Unit Occupational Therapy and Physical Therapy, Karolinska University Hospital, Stockholm, Sweden

**Keywords:** COPD, Personalised medicine, Telemedicine, User involvement, Video conference

## Abstract

**Background:**

Using participatory methods to engage end-users in the development and design of eHealth is important to understand and incorporate their needs and context. Within participatory research, recent social distancing practice has forced a transition to digital communication platforms, a setting that warrants deeper understanding. The aim of this study was to describe the experiences of, and evaluate a digital co-creation process for developing an eHealth tool for people with chronic obstructive pulmonary disease (COPD).

**Methods:**

The co-creation was guided by Participatory appreciative action and reflection, where a convenience sample (n = 17), including persons with COPD, health care professionals, relatives and a patient organization representative participated in six digital workshops. User instructions, technical equipment, and skilled support were provided if necessary. Workshops centred around different topics, with pre-recorded films, digital lectures and home assignments to up-skill participants. Process validity, experiences and ownership in the co-creation process were evaluated by repeated respondent validation, member checking, questionnaires and by assessing attendance. Data was analysed quantitatively or qualitatively as appropriate.

**Results:**

The co-creators were in general satisfied with the digital format of the workshops. Mean attendance and perceived engagement in workshops was high and the experience described as enjoyable. Engagement was facilitated by up-skilling activities and discussions in small groups. Few had used digital communication previously, and feelings ranging from excitement to concern were expressed initially. Technical issues, mainly audio related, were resolved with support. At completion, skills using equipment and digital platform surpassed expectations. Few disadvantages with the digital format were identified, and advantages included reduced travel, time efficiency and reduced infection risk.

**Conclusions:**

Experiences of digital co-creation were overwhelmingly positive, despite initial barriers related to computer naivety and use of digital equipment and platforms. The high level of satisfaction, engagement, attendance rates, and agreement between individual and group views suggests that a digital co-creation process is a feasible method. Several important success factors were identified, such as the provision of information and education on discussion topics in advance of workshops, as well as the smaller group discussions during workshops. The knowledge gained herein will be useful for future digital co-creation processes.

## Introduction

A progressive digitalization of public health is emerging, with electronic health (eHealth) advocated as a means to support and enable public health without compromising quality, accessibility, efficiency, and equity [1]. eHealth encompasses computers, smartphones, tablets, mobile sensors and wearables, apps, social media and online information, which can be used to personally monitor and inform health, as well as for communicative interaction, and the collection, management and utilization of health data [2]. In particular, eHealth may facilitate greater control of, and a more active role in personal health [2]; its potential for self-management of chronic diseases, including diabetes, cancer, cardiovascular-, and respiratory disease across populations is promising [3, 4]. For effective self-management of chronic disease using eHealth products or systems, the understanding and incorporation of intended users’ needs and context appears to be a pre-requisite [5]. For this purpose, the engagement of representatives of the specific population targeted (end-users) in the development and design of eHealth is increasingly promoted, and often approached using, for example, participatory methods or co-creation [6, 7]; the latter defined as ‘collaborative public health intervention development by academics working alongside other stakeholders [8].

Chronic obstructive pulmonary disease (COPD) is one of the most common chronic diseases, with substantial ensuing risk of morbidity and mortality [9]. Pulmonary rehabilitation is a comprehensive intervention including, but not limited to, tailored physical exercise, education and behaviour change [10]. Conclusive evidence suggests that pulmonary rehabilitation benefits patient-orientated outcomes in COPD such as improved physical capacity [11, 12]. Nevertheless, access to pulmonary rehabilitation is limited to a minority of the COPD population [13, 14], and improved access has been highlighted as a top priority for the coming decade. eHealth as a means to deliver pulmonary rehabilitation is a promising way to increase access, with increased reach [15], and level of physical activity indicated [16, 17]. However, recent systematic reviews of the effects of eHealth for the management of COPD have shown inconsistent results due to the high variability between studies and the interventions evaluated [18–27]. Larger studies, with high-quality study design, and where the eHealth intervention has been developed in co-creation with end-users, are warranted.

With the ambition to increase access to evidence-based treatment for people with COPD, our research group began preparations for a project to develop a novel eHealth tool, which later was named Min KOL (Eng: My COPD), in co-creation with prospective end-users and stakeholders through a series of workshops. Early in the project, the COVID-19 pandemic, and subsequent social distancing practice to protect risk groups, forced a transition of the entire co-creation process onto a digital communication platform. Digital co-creation has been suggested as an opportunity for wider inclusion and several potential advantages have been mentioned [28]. Although digital co-creation processes are relatively uncommon within research to date, this will likely change in line with the ongoing digitalization of most areas of society and therefore requires deeper understanding. The aim of this study was to describe the experiences of, and evaluate a digital co-creation process for developing an eHealth tool for people with COPD.

## Methods

### Study design

The procedural components of the co-creation process for the eHealth tool adhered to recommendations and principles outlined by Leask et al. [8]. The co-creation process was guided by Participatory appreciative action and reflection (PAAR). PAAR is a participatory method used to generate knowledge by exploring and developing success factors and facilitating collective learning, pluralism and reflection [29].

### End-users and co-creators

A convenience sample of co-creators were approached and invited to participate in the digital co-creation process and recruited through the authors' professional networks, primary- and specialist care registers, and the national patient organization. Co-creators were defined as persons with COPD, that is, the end-users [8], but also stakeholders involved in the tool's later implementation and use. Criteria for eligibility as end-user co-creators were (1) a COPD diagnosis according to the Global initiative for chronic obstructive lung disease (GOLD) [9], (2) ability to use a smartphone or a tablet, and (3) living within public health care regions Västerbotten or Stockholm, Sweden. Further representatives of stakeholders perceived to be involved in the use and implementation of the eHealth tool, and who were invited to take part in the workshops as co-creators, were physiotherapists, a COPD-nurse and a physician experienced in pulmonary rehabilitation within primary care or specialized care, relatives (of both sexes), and a representative from the national patient organization. Five persons with COPD and one relative to a person with COPD declined participation due to the group setting (n = 1), unknown reasons (n = 2) and due to fears of not being able to master the digital communications platform and associated technical equipment (n = 3), while three of the health care professionals approached declined due to the added workload. The sample was composed with end-user co-creators constituting a majority and aiming for maximum variation to encompass a wide spectrum of perspectives [30], with regards to sex, age, disease severity, ethnicity, and urban or rural living. Researchers (SL, AT and PS) from Umeå University and Karolinska Institutet took part in and moderated the workshops. In the first workshop a software developer moderated one of the small discussion groups. The moderators (SL, AT, PS) had previous experiences of conducting qualitative interviews, co-creation workshops and/or digital teaching. The moderators designed the study, the content and structure of the workshops and home assignments based on guidelines for co-creation [8]. Background data of co-creators, including age, sex, pulmonary function, symptoms using the COPD assessment test [31], ethnicity, and level of physical activity using questions from the Swedish National Board of Health and Welfare [32] were collected as appropriate (Table 1).

**Table 1:** Characteristics of co-creators

Characteristics^a^	Persons with COPD (n = 10)	Relatives (n = 2)	Health care professionals (n = 5)^b^
Age, years	71.1 ± 10.8	74.5 ± 6.4	n/a
Women	6 (60)	1 (50)	4 (80)
*Public health care region*			
Region Västerbotten	5 (50)	2 (100)	2 (40)
Region Stockholm	5 (50)	0	2 (40)
Other	0	0	1 (20)
Living rural	4 (40)	2 (100)	n/a
*Employment*			
Occupational pension	7 (70)	2 (100)	1 (20)
Disability pension	2 (20)	-	-
Gainful employment	1 (10)	-	4 (80)
Years within profession (min–max)	n/a	n/a	20 ± 14 (5–36)
Time since COPD diagnosis, years (min–max)	9.6 ± 8.2 (1–24)	n/a	n/a
FEV_1_, % predicted (min–max)	49 ± 24 (20–91)	n/a	n/a
FEV% (min–max)	51 ± 17 (22–70)	n/a	n/a
COPD assessment test (min–max)^c^	18 ± 10 (4–33)	n/a	n/a
Moderate-vigorous intensity physical exercise ≥ 30 min/week	5 (50)	n/a	n/a
Low intensity physical activity (≥ 10 min bouts) ≥ 30 min/week	7 (70)	n/a	n/a

COPD: Chronic obstructive pulmonary disease; FEV_1_: Forced Expiratory Volume in one second; FEV%: Forced Expiratory Volume (FEV_1_/FVC)

^a^Values are mean ± standard deviation, SD or number (percent, %)

^b^Including the patient organization representative

^c^Scores ranging from 0 to 40, with higher values indicating more symptoms

### Digital video communication

The workshops were conducted through the digital video communication platform Zoom (Zoom Video Communications, Inc 2021). The co-creators took part in the digital workshops from a location of their choice. A simple user manual with written instructions on how to download, access and use the video communication platform was developed and provided, as well as technical equipment (headsets, tablets, web cameras, mobile surf) when needed.

Three technical meetings were hosted before workshops commenced, where co-creators were encouraged to access the platform and receive an introduction to basic functions required in the workshops, for example, how to turn on/off audio- and video functions, change the view and ask questions. Two skilled technical staff, from the department of information and communication technology (ICT) services and system development at Umeå University, hosted and took part in the technical meetings. The ICT-staff were also present at the beginning of each, and available between each workshop via e-mail and telephone, to solve any issues related to the technical equipment or to the digital communication platform.

### Workshops

**Fig. 1:**
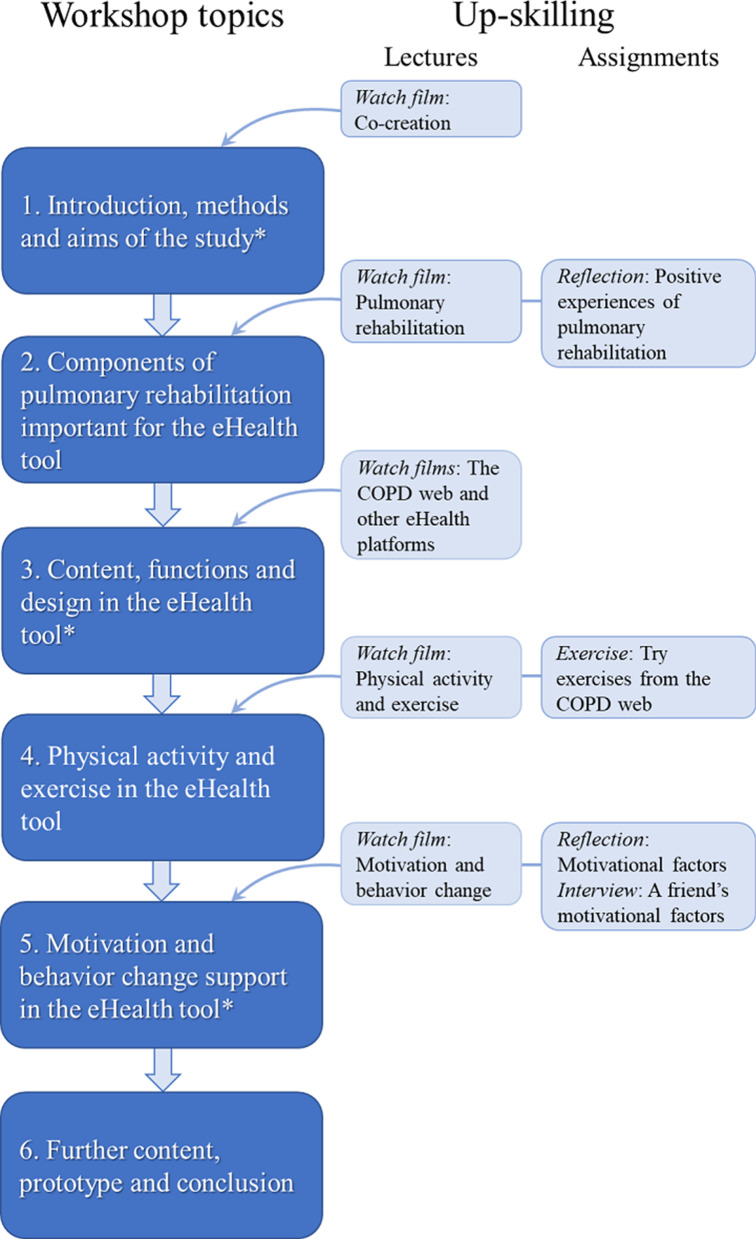
Overview of the co-creation process with workshop topics, and lectures and assignments used for up-skilling. *Denotes workshops where health care professions (physiotherapists, nurse, physician) and the representative from the national patient organization participated

Workshops, with discussions and practical tasks, are used within participatory methods to produce and collect reliable and valid domain specific data [33]. The purpose of the workshops was to explore population specific features and requirements regarding content and design and to develop the eHealth tool accordingly. A series of 6 workshops were hosted by the researchers (Fig. 1). Each workshop lasted no more than 2 h and had regular breaks to optimize concentration and reduce risk of digital fatigue [34]. The workshops were scheduled to start between 2–3 pm, since symptoms of COPD often present as less severe in the afternoon compared to the morning [35]. The health care professionals and the patient organization representative were invited to participate in 3 workshops only (workshop 1, 3 and 5), as we anticipated that daytime meetings could hinder participation. Furthermore, upon own request, the patient organization representative also participated in the last workshop. The workshops were spaced 2–4 weeks apart to allow time for the moderators to prepare or follow-up on ideas and suggestions arisen in workshops or in evaluations, and to not cause an unnecessary burden on the co-creators. Between the workshops, the moderators also discussed how to facilitate engagement by the co-creators in the following workshops. All information and contact with co-creators occurred via telephone or e-mail, with information and films provided to co-creators in advance of workshops and placed on a shared drive accessible to all co-creators.

The workshops had an iterative structure, and contained reflection, activity, planning and evaluation. Except for the first, each workshop began with a short reflection whereby a summary from the previous workshop discussions and outcomes were presented and its content and accuracy refined by the co-creators. The agenda and aims of the day’s activities were outlined, which included presentations from invited speakers, as well as whole or small group discussions, which constituted a majority of the workshops. For the small group discussions (20 to 40 min) moderators divided the co-creators into 3–4 groups, which varied between workshops. Sex, age, type of co-creator and level of engagement was considered when allocating co-creators to groups to promote interaction. Towards the end of each workshop a plan for the next workshop was presented and corroborated with co-creators, including a home assignment that could comprise watching a film, reflecting on a particular question, or interviewing a friend or relative. After each workshop, all co-creators were asked to complete a digital questionnaire. Moderators completed a separate digital questionnaire related to the co-creation process.

Important aspects and components of co-creative design that were incorporated and encouraged were co-creator ownership to increase creativity and productivity [36]. The equal standing of all co-creators, important for ownership, was affirmed and promoted by moderators in each workshop. Rules and responsibilities were negotiated by co-creators, for example, when and how to speak, and to respect each other’s opinions. Co-creators also took part in aspects of the planning process and decision making at workshops, for example, by viewing and commenting on the following workshop’s topic. Furthermore, all co-creators were “up-skilled”, i.e. received relevant information and knowledge necessary for meaningful participation and informed decision making in the workshops. By up-skilling, the capacity and capability of co-creators can increase [37] and potentially result in more innovative and meaningful solutions [38]. Up-skilling was conducted by watching pre-recorded films or digital subject-area expert lectures, or by browsing specific web-based platforms for information in between and in preparation for workshops. Most up-skilling activities took place between workshops. This freed up time in the workshops for interactive activities and discussions that facilitated co-creator engagement. Activities in the workshops centred around pre-planned topics that were considered important for the development of the eHealth tool (Fig. 1). The initial workshop focused on introducing the co-creators and to get to know each other, with the aim to facilitate interactions in the digital workshops. A running topic throughout the workshops, was the creation of a name and a graphical element to represent the eHealth tool to enhance ownership.

### Measurements

Data on characteristics of co-creators were collected using structured questions during a phone call prior to the co-creation process.

All group discussions in the workshops were documented through audio recordings and notes taken by the moderators. Moderators kept a log of all further communication related to the project, including mail- and telephone conversations. An evaluation of process validity was embedded throughout the co-creation process to ensure that outcomes were representative of the co-creators’ opinions and suitable, tailored and valid for end-users [8]. The evaluation was conducted by “respondent validation”, where within each workshop moderators’ summaries of the small group discussions were presented to the whole co-creator group and refined. The evaluation was also conducted by “member checking”, whereby the co-creators were asked to reflect on discussions and conclusions conducted in the previous workshop.

The co-creators’ experiences and ownership were evaluated through a digital questionnaire distributed at the end of each workshop, and at the completion of the workshop series. In the questionnaires at the end of each workshop, open questions were used to explore perceptions of the information provided and experiences of taking part, as well as, ways to carry across their views in the digital workshops. Furthermore, multiple choice questions were used to assess perceived agreement between own views and group conclusions (no-, part-, near complete-, complete agreement) and engagement in the workshops (not-, partly-, completely engaged). In the questionnaire at workshop series completion, experiences of the advantages and disadvantages of conducting the workshops digitally, the home assignments (up-skilling), and handling the technical equipment necessary were explored. Another aspect of ownership is loyalty [8, 39], which was evaluated by assessing the attendance rate of the co-creators in the study.

Moderators completed a separate digital questionnaire at the end of each workshop, and at the end of the workshop series. With the purpose to continuously develop and evaluate the co-creative process in the workshops, they answered questions related to facilitating factors and barriers, and the co-creators’ engagement in the co-creative process.

### Data analysis

Co-creator characteristics, attendance and response rates were summarized using descriptive quantitative statistics. The co-creators’ responses in the questionnaires were summarized using descriptive qualitative analysis. First the responses were read through, and sorted into themes: experiences of the workshops, experiences of knowledge up-skilling and engagement, and experiences of the digital format of the workshops. Each theme was then described in text, with illustrative quotes from the evaluations. The same procedure was conducted for the moderators’ evaluations, notes and summaries related to the themes: the moderator role, and facilitators and barriers for engagement. The qualitative analysis was inspired by thematic analysis [40]. The transcribed audio recordings were read through, and indications of technical issues noted. The qualitative and quantitative findings were interwoven and presented together in the results section. Two of the moderators analysed the quantitative (AT) and qualitative (SL) data and described the results, while the third moderator (PS) and two researchers (KW and AH) who were not involved in moderating the workshops critically reviewed the results.

All authors are registered physiotherapists, with extensive experience in research and clinical work within exercise training for people with COPD (PS, KW) or older adults (AT, AH). The authors also have experience working with and developing eHealth (SL, PS, AH, KW) and in the development of eHealth tools for people with COPD using co-creation (SL, KW). Finally, the authors have expertise conducting research studies utilising qualitative (SL, AH), quantitative (AT, KW, AH) or mixed methods (SL, KW, AH).

## Results

### Co-creators

The study included 17 co-creators in total, 11 women and 6 men. The end-user co-creators were aged between 51 and 87 years, and were diagnosed as GOLD 2–4. Three participants receiving long-term oxygen treatment. All co-creators except one were born in Sweden. Characteristics of the co-creators are presented in Table 1.

### Loyalty

The median attendance rate in workshops was 89% in persons with COPD, while remaining co-creators (relatives, health care professionals, patient organization representative) had 100% attendance rate. Reasons for not attending workshops were related to personal engagements such as attending a funeral or work related. In the last two workshops, most non-attendance occurred (n = 5), and reasons included computer failure (n = 1), forgetfulness (n = 1), acute illness (n = 1), unknown (n = 1), and deceased (n = 1).

### General experiences of the process

The co-creators were in general satisfied with the structure and content of the co-creation process. Participation in the workshops was described as interesting, nice, informative and rewarding. The structure of the process was considered clear and varying, and varying the activities between larger and smaller groups was appreciated. The length of the workshops and the time between workshops were generally perceived to be adequate. However, a wish for more time for discussions in the smaller groups was expressed, since this setting was highlighted as particularly valuable for discussions. The information provided in the beginning of and between the workshops, including the films with pre-recorded lectures by the invited subject matter experts, was described as interesting, informative and clear.

Good with collated instructions and notes. Easy to look back on.

(Co-creator, final evaluation)

### Process validity

Although opinions initially varied in the discussions, the co-creators were able to come to an agreement on issues.

Interesting to see that we have such different starting points. But we nearly always reach the same end point.

(Co-creator, workshop 4)

**Fig. 2:**
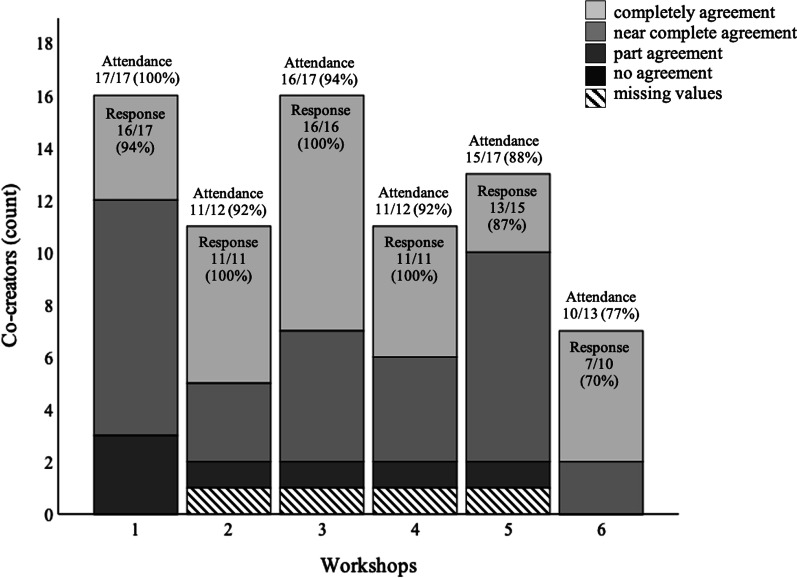
The co-creators’ perceived agreement between individual views and group conclusions. All co-creators were invited to workshops 1, 3 and 5, while only persons with COPD and relatives were invited to workshops 2, 4 and 6. The patient organisation representative also attended workshop 6. Attendance = attendance rate (of invited co-creators); Response = response rate (of attending co-creators)

Most co-creators rated a complete or near complete agreement between their individual views and the conclusions from the group discussions (Fig. 2), suggesting that process validity was high.

### Experiences of shared knowledge

To get information about the aim of the workshops and the project, as well as about the expected results and future plans was considered important by the co-creators. A wish for more workshops to test the eHealth tool before its release was requested. The co-creators also expressed a desire to participate in more research studies in the future.

The up-skilling tasks and home assignments between the workshops was considered important for their engagement in the workshops.

I think it is important with some preparation with for example the film so that the workshop itself gets going easier.

(Co-creator, workshop 5)

In the co-creation process, it was the co-creators' experience that new knowledge about COPD was gained. New knowledge about aspects of pulmonary rehabilitation, especially the importance of physical exercise and how to perform it was mentioned. Information about COPD and advice for promoting health was appreciated, as well as new knowledge about which interventions they were entitled to.

The knowledge about COPD that I did not have before. I feel especially grateful for that and know more what I can ask for at the health care centre.

(Co-creator, final evaluation)

### Engagement

**Fig. 3:**
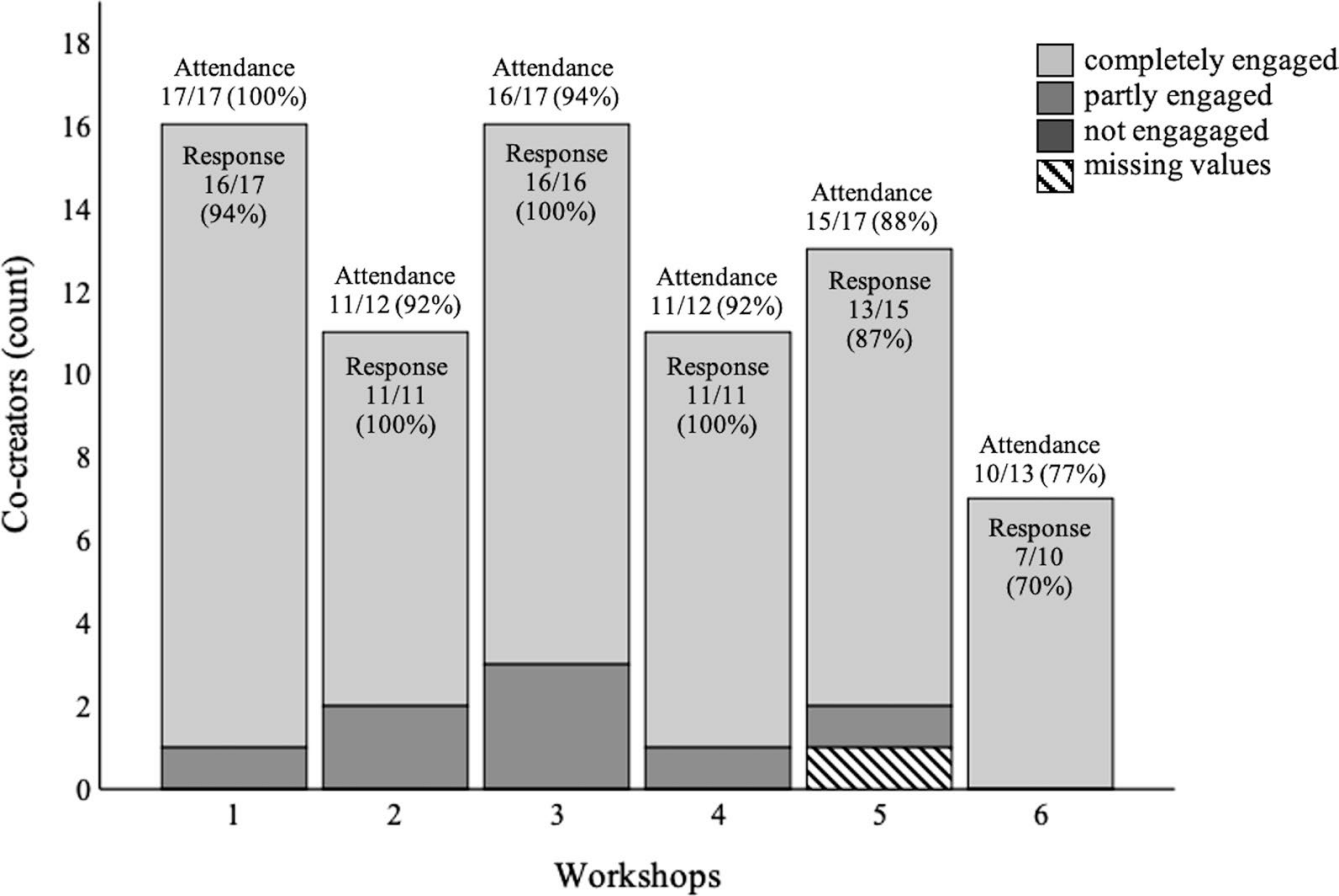
The co-creators’ perceived engagement in the workshops. All co-creators were invited to workshops 1, 3 and 5, while only persons with COPD and relatives were invited to workshops 2, 4 and 6. The patient organisation representative also attended workshop 6. Attendance = attendance rate (of invited co-creators); Response = response rate (of attending co-creators)

The perceived engagement in workshops was by most co-creators rated as high (completely engaged) throughout the workshop series (Fig. 3). The co-creators felt able to contribute with their opinions in the workshops and discussions. In addition, they found it interesting to engage with other people with knowledge of what living with COPD entails, and to share experiences and thoughts. The co-creators described the discussions as creative and constructive, that the communication in the group was good, Thus, the co-creators believed that they complemented each other and they seemed to enjoy getting to know each other. A respectful and relaxed climate in the group was according to the co-creators valuable for their engagement. To feel welcomed, seen and heard made expressing views easy.

We talked to each other and gave feed-back. Everyone talked in the small group and it was a good and friendly atmosphere. It was also easy to engage in the large group. If I thought something about an issue, I would express my opinion or idea.

(Co-creator, workshop 1)

### The digital format of the workshops

In total, nine co-creators borrowed technical equipment, 2 web-cameras, 9 headsets, and 3 tablets. Mobile surf was supplied to one co-creator. While the health care professionals and patient organization representative had all used the video communication platform previously, of the end-user co-creators and relatives only one had previous experience.

The digital format of the workshops was in general experienced as positive by the co-creators. Nevertheless, prior to the first workshop, a few co-creators described feeling excited and a bit nervous about participating in the digital workshops, due to low computer skill and unfamiliarity with the digital technology.

New and a little gruesome before with the digital that I do not have much experience of.

(Co-creator, final evaluation)

However, as the workshops commenced, surprise and astonishment about how well they managed the digital technology was expressed.

Since I have no computer experience it was exciting to see if I would keep up. But it was just to talk.

(Co-creator, workshop 1)

Technical issues were noted during all workshops, in total on 51 occasions (5–11 per workshop). Most issues related to difficulties using technical equipment or functions in the digital platform, for example turning the sound on or off or positioning the microphone correctly (n = 17), using screen sharing or adjusting screen view (n = 12), moving into break-out groups or leaving the meeting (n = 4); and resolved in the workshop by moderators, co-creators or the ICT-staff. The technical issues between workshops concerned downloading and accessing the video communication platform and sound issues, which the ICT-staff resolved by telephone support. Still, a few major efforts were needed by the ICT-staff; one home visit and one remote control of a computer to download and install the video communication platform, and support to one participant by telephone and email to resolve persistent sound issues. In total, the ICT-staff provided 13 h of support throughout the digital co-creation process. Technology and support were also mentioned by the co-creators in the evaluations. The written instructions on how to install and use Zoom, and the option to borrow technical equipment was appreciated. As the workshops progressed, an increase in technical capability and confidence in participating in digital meetings were described.

Not many disadvantages of performing digital workshop were identified, although technical issues were mentioned. However, in comparison to digital workshops, real-life meetings were perceived to offer better possibilities to “get to know the others”, to “talk in a different way”, and “probably more fun”.

In contrast, several advantages of performing digital workshops were mentioned, such as avoiding travel and parking, reduced infection risk, time efficient, simple, and the possibility to participate regardless of geography.

Many participants and also invited “guests” in the same place, even though they were spread out over the country.

(Co-creator, final evaluation)

### Development of the co-creative process

During the study, the moderators’ views of the importance of engagement in the co-creation process was affirmed. They perceived group belonging to be facilitated by the design of a graphical element for the eHealth tool. Technical issues, for instance, reduced sound, were viewed as the largest barrier for engagement. Further, the workshops were perceived to have an open and pleasant climate, where everyone got to speak. The moderators were responsible for noticing if a co-creator had been quiet for a while and to encourage inclusion in the discussion. It was experienced as stressful to both facilitate the discussion and take notes at the same time, however the co-creators assisted the moderators in noticing a problem or if someone wanted to say something. The moderators described the need for a different approach in digital workshops compared to real-life meetings. They viewed their role as moderators important in the digital workshops, especially in order to get a flow in the discussions.

Digital implementation makes it easier for participants to delay their answer or share their thoughts, the forum demands that the situation is more controlled.

(Moderator, workshop 5)

The moderators found it difficult to find balance in how much to structure the discussions in the digital format. In the initial workshops they perceived that much of the discussions in small groups occurred through them, and that the co-creators for the most part only answered questions addressed directly to them by the moderator, rather than openly discussing with each other. This was by the moderators viewed as a great responsibility.

It still feels a bit stressful that the group’s conversation depends a lot on me as leader, it is important to bring out the group’s opinions and experiences, you do not want to miss anything because you were not able to extract from the participants that which was important to them.

(Moderator, workshop 2)

It was therefore perceived that semi-structured questions or work tasks were needed to stimulate discussions, which would aid the moderators’ in facilitating the discussion by asking questions and distributing the word between co-creators. The moderators expressed a wish for discussions and interactions to be freer and more spontaneous, and when that happened the moderators’ role was perceived as more enjoying, easy and relaxing. The responsibilities were divided between moderators and kept constant throughout the co-creation process, which was experienced as positive and reassuring. As the moderators grew more comfortable in their roles they felt that their skills complemented each other’s.

Overall, it was the moderators’ experience that while all co-creators contributed, engagement was especially high in the smaller groups. Thus, the small group discussions were considered pivotal for engagement, while discussions were inhibited in the large group. Consequently, when planning the workshops, the smaller group discussions were prioritised and allocated sufficient time. The small group discussions were often described as slow to start, but became more active as the co-creators got more used to each other and the process.

Moderators viewed co-creators with more experiences related to the topic of the workshop as more active than co-creators with no or little experience. Consequently, well prepared workshops and clarity about the method, topics, home assignments and the structure of the workshops was described as important for engagement. The home assignments and question time with subject matter experts were seen as a facilitator for discussions. In addition, the moderators viewed the varied background and competence of the co-creators as an advantage that stimulated the discussions. However, early in the process it was perceived that the health care professionals held back in the discussions to allow the other co-creators room to talk.

The health care professionals seemed a little hesitant, as if they were primarily letting persons with COPD take their place.

(Moderator, workshop 1)

Consequently, when relevant for the topic, the smaller discussion groups were formed with health care professionals only in one group.

## Discussion

### Principal results

To the best of our knowledge, this is the first study to report experiences from a digital co-creation process in the development of an eHealth tool for people with COPD. The experiences were overwhelmingly positive. The co-creators were satisfied with the content and structure of the co-creation process, and the smaller group discussions were considered an especially valuable setting for discussions. While the workshops had good attendance rates throughout the workshop series, the last workshop seemed to have the lowest attendance rate. Engagement in workshops appeared high throughout and believed to be facilitated by preparations which included providing co-creators with information of the content in workshops and discussion topics in advance, and the creation of a respectful and relaxed climate in the workshops. Most co-creators reported a complete or near complete agreement between their individual views and the conclusions in the group at workshops. Some advantages of the digital format included avoidance of travel and parking problems, reduced infection risks, and the geographical diversity of participants. Barriers included computer naivety and limited experience of digital equipment and video communication platforms. Despite initial computer naivety and apprehension, the digital format of the co-creation workshops was experienced as positive, and the co-creators were surprised at how well they managed the technique after all. Some technical issues were noted throughout, by moderators and co-creators alike, which could be resolved in the majority of cases.

### Interpretation of findings

Recruitment, support and training have been pointed out as key factors for meaningful involvement of patient and public representatives [8, 41]. Convenience sampling is the most reported method to identify patients in studies with patient engagement [42], and was used to recruit not only participants with COPD, but also relative-, health care-, and patient organization representatives in this study. In the recruitment of participants, a balanced socio-demographic representation is important to consider [41]. We strived to include co-creators with varied perspectives, and believed that sex, age, disease severity, ethnicity, and urban or rural living, could influence preferences for an eHealth tool. Another aspect to consider in the recruitment process is the potential participants’ contribution to the group dynamic. Participants who domineer the discussions, or remain passive, may influence the group dynamics negatively [43]. This study found, that in a digital setting the moderators had an important role in engaging the participants. The importance of a moderator, whose role is to allow everyone to be heard, has been confirmed elsewhere [43]. A dedicated contact person who can answer questions between or before meetings has been suggested to support and facilitate engagement [43]. In our study, one researcher was responsible for most email and telephone contacts with participants, and was therefore often contacted by the participants if questions arouse or if unable to attend a workshop. Sufficient time to build reciprocal relationships, for discussions [42, 43], and repeated meetings to build group dynamics [43] have also been reported important for engagement. In this study, although participants perceived the workshops to have an adequate time-frame, more time allocated for discussions, as well as more workshops were wished for. Meanwhile, at the two last workshops attendance was attenuated, possibly a result of decreased motivation. Motivation has been described as a driving force for people with COPD and health care professionals to participate in a co-creation process [44]. Training, or up-skilling participants, has been reported as important for engagement and creativity in many studies [8, 41, 43, 45], which our results supports. In the process planning of this study, several steps were taken to facilitate knowledge development in the participants, such as information on the aim of the process and the participants’ role [43, 45], education in the topics of the workshops, and adequate time for preparations [43]. According to Leask, et al. [8], up-skilling is an important part of ownership. In our study, ownership was further facilitated by promoting the equal standing of co-creators and moderators alike, and including co-creators in planning and decision making. The positive experiences by the co-creators and moderators suggest that ownership is an important feature in co-creation. In people with COPD and health care professionals, feelings of responsibility have been reported as a driving force in co-creation [44].

Computer literacy is an important part of eHealth literacy, and includes the ability to use computers to solve problems and to adapt to new technologies and software [46]. The participants in this study experienced the digital format of the workshops as positive. In Sweden, more than 90% of the population in most age groups have access to the internet at home [47]. Furthermore, in a cohort of people with COPD in Sweden, over 90% had access to the Internet and 85% almost used it every day [48]. This provide good opportunities for conducting digital workshops. In groups with chronic respiratory diseases, positive views towards eHealth tools and high probabilities of using such tools have been shown [48, 49]. Still, in this study technical issues were considered a barrier for engagement. In the recruitment process, three potential participants declined participation in our study because of a fear to not master the digital format. Comfortability with digital tools have been shown to affect usage of digital tools in people with COPD [50]. The co-creators with COPD in this study all owned and had used a smartphone or a tablet previously, however, the level of computer skills varied. Some had low computer skills and were unfamiliar with using email for communication and needed assistance, while others had higher computer skills and were accustomed to using Zoom or other digital communication platforms. A Swedish study of older persons with chronic diseases, reported a lack knowledge and fear of using new technology, but also an interest and a willingness to learn more about digital tools [51]. Similar to the participants’ experiences in our study, a transition over time was shown in a telemonitoring study with people with COPD, where initial insecurities of using the technical equipment gradually eased as confidence grew [52]. In that study, more supervised testing of the equipment was requested to improve confidence [52]. This was implemented in our study, where opportunities to test the digital platform before the co-creation process started were provided. Another successful approach was having ICT-staff who provided ongoing support to participants with technical issues in the workshop. This allowed the workshop to continue without interruptions. The technical support would have been easier if all participants had been provided with and used the same equipment.

### Lessons learnt

The lessons learnt in this study were many. Using recommendations for planning the co-creative process, with adaptions for a digital forum, seemed a useful strategy as participants appreciated the structure and content. Keeping the digital workshop sessions relatively short may require a greater need to prioritise interactive activities such as discussions in the workshops. We found pre-recorded films between workshops very effective for up-skilling the co-creators, albeit time-consuming to plan and prepare. In the workshops, most time was allocated for small-group discussions, which were also found to aid discussions and engagement. Moderators may need to take a more semi-structured approach, with clear questions and activities prepared, when moderating digital workshops compared to real-life workshops. Furthermore, to have access to information and conclusions between workshops were appreciated by the co-creators. The time and efficiency for the provision of technical support, for example guides on how to download and use programs, and opportunities to practice were aided by including ICT-staff in the project. With support even co-creators with low computer skills were able to participate in the workshops.

### Strengths and limitations

The adherence to guidelines for conducting and reporting results from co-creation studies [8], and the use of PAAR is a strength of this study. By structuring and planning the study accordingly, aspects important to the quality of the co-creation process were incorporated. Between workshops, the moderators continuously evaluated and discussed ways to facilitate engagement in activities, home assignments and by using different group constellations. According to the PAAR approach, activities and responses were designed to elucidate and build on positive experiences and good examples [29]. Triangulation [53] between authors also strengthen the trustworthiness of the study. During the planning of the study, it was decided that to reduce workloads of the health care professionals and the patient organization representative, they should only take part in three of the workshops. Nevertheless, at the end of the workshop series the patient organization representative requested permission to participate in the last workshop. Since the participant was deemed to contribute to the group discussions the request was approved. In the recruitment, a variation in perceptions were strived for, including the ethnicity of participants. However, only a limited variation in ethnicity was achieved, with only one participant born outside Sweden. The difficulty in finding and recruiting individuals born outside Sweden has been reported in a previous study [54]. Including individuals with different ethnicities would have enriched the study and is recommended in future research on the subject.

## Conclusions

In this study, the experiences of the digital co-creation process in the development of an eHealth tool for people with COPD were overwhelmingly positive, despite barriers related to computer naivety and use of digital equipment and platforms. The level of satisfaction, engagement and loyalty throughout the workshop series, as well as the level of agreement between individual and group views suggests that a digital co-creation process is a feasible method. Although many advantages with the digital process were identified, such as, avoidance of travel and risk of infection, highlighted barriers included computer nativity and limited experience of digital equipment and video communication platforms. Several important success factors were identified, such as the provision of information and education on discussion topics in advance of workshops, as well as the smaller group discussions during workshops. The knowledge gained herein will be useful for future digital co-creation studies.

## Data Availability

The data used during the current study are available from the corresponding author on reasonable request; however, it may only be shared in part (to ensure confidentiality).

## Abbreviations

COPD: Chronic obstructive pulmonary disease
FEV_1_: Forced expiratory volume in one second
GOLD: Global initiative for chronic obstructive lung disease
ICT: Information and communication technology
PAAR: Participatory appreciative action and reflection
